# Meta-Analysis of Elderly Lower Body Strength: Different Effects of Tai Chi Exercise on the Knee Joint-Related Muscle Groups

**DOI:** 10.1155/2021/8628182

**Published:** 2021-12-22

**Authors:** Yuan Yang, Jia-hui Li, Nan-Jun Xu, Wei-Yi Yang, Jun Liu

**Affiliations:** ^1^The Second Affiliated Hospital of Guangzhou University of Chinese Medicine, Guangzhou, China; ^2^Bone and Joint Research Team of Degeneration and Injury, Guangdong Provincial Academy of Chinese Medical Sciences, Guangzhou, China

## Abstract

**Objective:**

To determine whether lower body strength such as keen extension and flexion strength may be improved by Tai Chi exercise in older adults from the perspective of evidence-based medicine.

**Methods:**

Databases of PubMed, Embase, and Cochrane Library were searched up to July 1, 2021. Randomized clinical trials are adopted to compare Tai Chi exercise with sedentary behavior or other low intensity exercise in terms of influence on lower body strength rehabilitation, especially keen extension and flexion strength in people aged over 60. A meta-analysis was performed to discuss outcomes of lower body strength, knee muscle strength, and knee extension/flexion strength.

**Results:**

A total of 25 randomized trials involving 1995 participants fulfilled the inclusion criteria. (1) Tai Chi exercise significantly improved elderly lower body strength (−0.54, [−0.81, −0.28], *p* < 0.00001, *I*^2^ = 74%), but there was no differential improvement in the strength of the knee joints (0.10, [−0.02, 0.23], *p*=0.11, *I*^2^ = 34%). (2) Elderly individual lower body strength declined with age, while this trend was suppressed by Tai Chi exercise (−0.35, [0.14, 0.56], *p*=0.001, *I*^2^ = 70%). (3) Although Tai Chi exercise did no significantly improve the large muscle group of knee joint extensor like quadriceps femoris (3.15, [−0.69, 6.99], *p*=0.24, *I*^2^ = 26%), it showed marked enhancement to the strength of deep small muscle group of knee joint flexor (10.25, [6.90, 13.61], *p* < 0.00001, *I*^2^ = 0%). The heterogeneity might be caused by distinguished measurements of muscle strength. Therefore, Tai Chi exercise specifically enhanced some certain muscle strength of knee joints and improved muscle fitness rehabilitation as well as function activity for elderly.

**Conclusions:**

In this RCT meta-analysis, Tai Chi exercise has positive effects on lower body strength of elderly. Although no obvious improvement on the knee extensor is observed, it may be used as a rehabilitation treatment for training stable deep muscle groups to improve the knee flexion strength significantly.

## 1. Introduction

Tai Chi exercise is considered of high clinical value to rehabilitation of diseases. Combined with the latest research in bioinformatics and health informatics, it mostly involves the biological, biomechanical, and psychosomatic medicine in the elderly healthy research [1]. Classically, deficiency in lower muscle fitness in elderly individuals makes them vulnerable to muscle fatigue, mobility disorder, and physical injury [2]. However, whether Tai Chi can improve elderly muscular strength is still controversial. Experts hold distinguished attitudes towards the effects of Tai Chi exercise on lower body strength of the elderly. The argument focuses mainly on the following aspects: (1) Is it possible that the external force stimulation of Tai Chi actions causes changes of “functional adaptability” to bone tissue or muscular strength? (2) Is physiological mechanism of Tai Chi, compared with other physical activities, characteristically concentrated on its energy efficiency of local bone and lower body muscle tissue? (3) Do prescription factors such as the duration or frequency of Tai Chi exercise affect the function on lower body muscle strength or not?

As a low-moderate intensity traditional exercise, Tai Chi is likely to prevent falls and enhance walking ability of elderly. A meta-analysis [3] shows persuasive evidence of improved exercise ability such as balance, movement, gait speed, and muscle fitness of elderly life. Zhou et al. [4, 5] believe that Tai Chi effectively postpones the decline of muscle strength caused by aging, and there was a significant improvement in lower body muscle strength for elder Tai Chi practitioners. Yang and Liu [6] collected 66 studies and found that Tai Chi may improve health fitness indicators of middle-aged and elderly people. Research of multiple sclerosis patients [7, 8] shows that Tai Chi has a positive regulatory effect on balance, coordination ability, walking ability, and muscle fitness. It also emphasizes that the improvement on lower body muscle strength by Tai Chi is related to the adjustment of knee joint strength and range of motion. Another meta-analysis [9] of 31 studies points out that Tai Chi improves muscle strength and reduce the risk of falls by enhancing the elderly's cardiopulmonary function, immune capacity, mental control, flexibility, and balance control.

However, other reports confirm that Tai Chi mainly improves muscle flexibility, dynamic balance, and joint stiffness rather than muscle strength or endurance [10, 11]. For example, the New Tai Chi Rehabilitation Program [12, 13] is shown to have no significant effect on the lower body strength of female elderly patients with knee osteoarthritis. Song et al. [14] also argue that there is no significant difference in knee joint strength of elderly women with osteoarthritis after 12 weeks of 12-form Sun style Tai Chi exercise. Since Tai Chi exercise relies heavily on horse stance or single leg, knee joints are in a state of weight bearing for a long time. Practitioners with insufficient lower body muscle strength are highly likely to suffer from severe strain in their own knee joints and lower limb muscles, which harms patients' rehabilitation [15].

In summary, the effect of Tai Chi exercise on the lower body strength is inconsistent in different research works. These conclusions cannot be supported or proved as systematic and medical evidence. Consequently, whether Tai Chi exercise can improve muscle strength in the elderly needs further research from the perspective of physical fitness. Here, we analyzed the related literature in recent years to explain the influence of Tai Chi on the lower body strength of the elderly, which may provide scientific guide and practical reference for health promotion.

## 2. Methods

Our meta-analysis was performed according to the Cochrane Handbook.

### 2.1. Search Trials

We searched the databases of PubMed, Embase, and Cochrane Library up to July 1, 2021. The keywords of “Tai Chi,” “muscular strength,” “keen extension,” and “keen flexion” were used to identify published systematic reviews or meta-analyses evaluating the association between Tai Chi exercise and the change of lower body strength, keen extension, and flexion strength. Searching strategy for this meta-analysis is available in 3.1. The language was restricted to English, and the search was restricted to systematic reviews or meta-analyses published from January 1, 2000, to July 1, 2021. We identified original randomized clinical trials (RCTs) included in the systematic reviews or meta-analyses.

### 2.2. Inclusion Criteria

*Inclusion criteria* were as follows: (1) All studies were RCTs including keywords such as “random,” “randomized,” and “control.” (2) Trials enrolled adults older than 60 years without limitation to their gender, race, or health condition. (3) Intervention measure of the experimental group is Tai Chi exercise without limitation to style, learning methods, or training sites. For the control group, the intervention measures were none, low intensity exercise, routine treatment, health education, routine exercise, etc. (4) Trials provided data of quadriceps femoris and other lower body muscles strength. (5) All articles are in English. *Exclusion criteria* were as follows: (1) randomized trials without a control group; (2) repeated publication literature; (3) ambiguous results or data and failure to contact the author; (4) unavailable full text; (5) reviews, animal experiments, conference abstracts, case reports, etc.

### 2.3. Risk of Bias Assessments

According to the Cochrane Collaboration Network, the bias in each trial was evaluated by 7 items as follows: the randomization sequence generation, allocation concealment, blinding of participants and personnel, blinding of outcome assessment, incomplete outcome data, selective reporting, and other bias. We defined other bias as trials of muscle strength measured by unauthorized methods and trials in which baseline characteristics were not similar between different intervention groups.

### 2.4. Data Extraction

Two researchers (Yuan Yang, Jia-hui Li) independently extracted the following information from each study: lead author; publication year; country of origin; participant characteristics; type, time, and intensity of Tai Chi exercise; interventions of control group; baseline of lower body strength; and trial duration. Disagreements were resolved by consensus. If the trials had more than 2 groups or factorial designs and permitted multiple comparisons, we extracted only the information and data of interest reported in the original articles. If a meta-analysis provided data that we did not retrieve, we extracted those fracture data from forest plots of the meta-analysis and reviewed original articles to confirm whether the trials met our inclusion criteria. When those data were our outcomes of interest, we pooled them with the data from primary trials.

The lower body strength of participants was the primary outcome because lower body strength might lead to more serious consequences than other elderly muscle strength. The secondary outcomes were the knee muscle strength, keen extension, and flexion strength. Knee muscle strength was defined as total knee joints muscles when they occurred at all sites or when trials did not describe the types of muscles in detail. If a trial only reported the strength of participants with certain type of muscles, such as keen extension and flexion strength, we did not consider it to be knee muscle strength.

### 2.5. Statistical Analysis

In this study, RevMan 5.3 software (provided by Cochrane Collaboration Network) was used for data analysis. Mean ± SD were used to process the included data obtained by the same measurement method. Standard mean difference (SMD) and 95% confidence interval (95% CI) were used to process the data obtained by different methods of measurement or units. Estimates for HRs were weighted by ANOVA variance and computed by random effects modeling. Statistical heterogeneity was assessed using *I*^2^ statistics. The appropriate effect model for the research indicators was selected, the combined effect statistics were analyzed, and then the forest plot was drawn. The risk of publication bias was assessed by the funnel plot.

Solutions to heterogeneity in this analysis process are as follows: firstly, checking the original data and confirming that the fixed effect model converts into a random effect model with high heterogeneity; then, removing the original data items one by one or excluding the lower quality literature for sensitivity analysis to find the possible source of heterogeneity; lastly, carrying out subgroup analysis according to different muscle strength measurement methods and different parts of muscle strength index.

## 3. Results

### 3.1. Retrieved Studies

**Figure 1:**
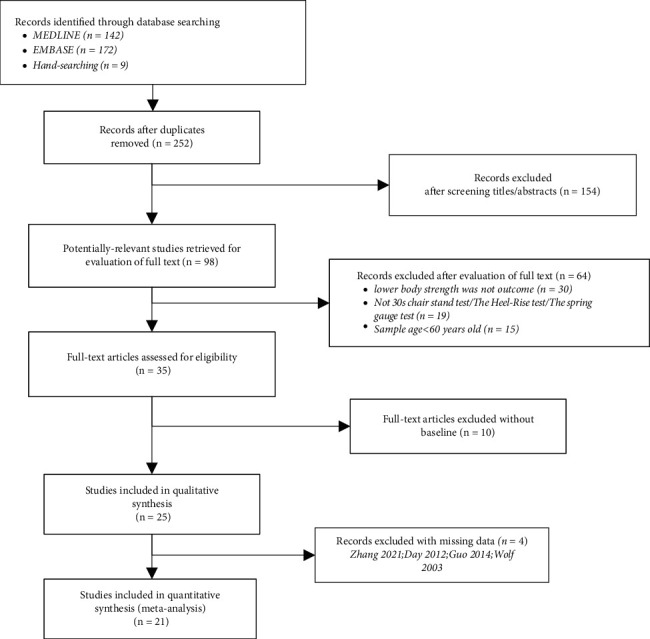
Study flow diagram of Tai Chi intervention on elderly muscular strength.

As shown in Figure 1, 252 unrepeated articles were reviewed according to the searching strategy (*searching strategy: PubMed [“tai ji” OR “t'ai ji” OR “tai chi” OR “t'ai chi” OR “tai chi chuan” AND (“muscular strength” OR “muscular endurance” or “flexibility”)] (142 articles)/Embase [(“tai ji”/exp OR “tai ji” OR “tai chi”/exp OR “tai chi” OR “tai chi chuan”/*exp *OR “tai chi chuan”) AND (“muscular strength”/exp OR “muscular strength” OR “muscular endurance” OR “flexibility”/exp OR “flexibility”) (172 articles)/manual retrieval with the articles closely related]*). Titles and abstracts of these records were screened for inclusion, and 154 articles which had nothing to do with the outcomes of this study were excluded. Full texts of 98 records were read, and 64 articles were deleted. Among these articles, 19 did not have their data collected by persuasive test (30 s chair stand test/heel rise test/spring gauge test), 30 mostly mentioned grip strength or upper body strength, and 15 did not meet the age of >60. 10 of the remaining 35 readings were not analyzed with baseline or control data. Four articles have missing data (Day et al. [16] with incomplete data of quad strength and no significant difference between left and right knee, Guo et al. [17] with incomplete baseline data of quadriceps extensors and hamstring flexor, Wolf et al. [18] with no data of limited changes of lower body strength, and Zhang et al. [19] with incomplete data of lower body strength). 21 RCTs with complete data were included for further analysis from the final 25 trials.

### 3.2. Characteristics of Included Studies

The relevant characteristics of 25 articles were listed according to the publication time sequence, as shown in Table 1, including 4 articles that did not carry out meta-analyses but included systematic reviews. Most trials did not group the participants by sex, and only a few focused on older women [14, 20–22]. All the training programs of Tai Chi exercise were set at ≥20 min, ≥2 days/week, and ≥6 weeks, most of which were set at 60 min/day and 2–4 days/week. In addition, the control group did sedentary or other low intensity exercise such as stretching and walking. Three methods (30 s chair stand test, spring gauge test, and isokinetic dynamometer test) were mentioned to detect muscle strength, which might be one of the factors evaluating the outcome indicators.

### 3.3. Outcomes of Literature Quality Assessment

**Figure 2:**
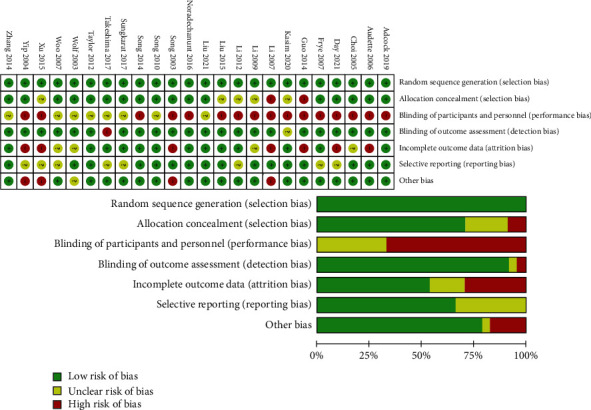
Risk of bias graph. We reviewed authors' judgements about each risk of bias item presented as percentages across all included studies. We defined other bias as trials of muscle strength measured by unauthorized methods and trials in which baseline characteristics were not similar between different intervention groups.

Figure 2 shows the assessment of the risk of bias. All studies were randomized. In addition, since this study mainly discussed the intervention effect of Tai Chi, which is a relatively popular traditional exercise in China, among the documents included in this searching, there are 12 trials from China. We have sorted out the main information for reference in Table 1. According to the risk assessment shown in Section 2.3, literature quality of these trials was evaluated and divided into three levels: “low risk,” “unclear,” and “high risk.” As shown in Figure 2, 20 trials described an adequate random sequence generation process, while other literature (Kasim et al. [23], Day et al. [16], Li et al. [5], Huang and Lin [24]) did not specify the random allocation method. As the intervention approach was Tai Chi exercise, it was difficult to achieve double-blind trials, so the risk bias of the whole assessment was high. While 17 trials described the methods used for allocation concealment, 5 trials (Audette et al. [22], Choi et al. [25], Guo et al. [17], Buto and Li [3], song et al. [14]) had incomplete outcome indicators with missing data of baseline testing or postintervention testing. In addition, there are unspecific descriptions of the withdrawal and missing visits in the trials (Day et al. [16], Woo et al. [26], Wolf et al. [18], Guo et al. [17], Li et al. [5], Song et al. [14], Xu et al. [20], Yip et al. [27]). To sum up, 4 trials (Day et al. [16], Wolf et al. [18], Xu et al. [20], Yip et al. [27]) were of low quality, 6 trials (Liu et al. [28], Song et al. [21], Sungkarat et al. [29], Taylor et al. [30], Woo et al. [26], Zhuang et al. [31]) were of high quality, and the others were of moderate quality.

### 3.4. Tai Chi Exercise and Lower Body Strength Risk

#### 3.4.1. Analysis of the Effects before and after Tai Chi Exercise on Lower Body Strength

**Figure 3:**
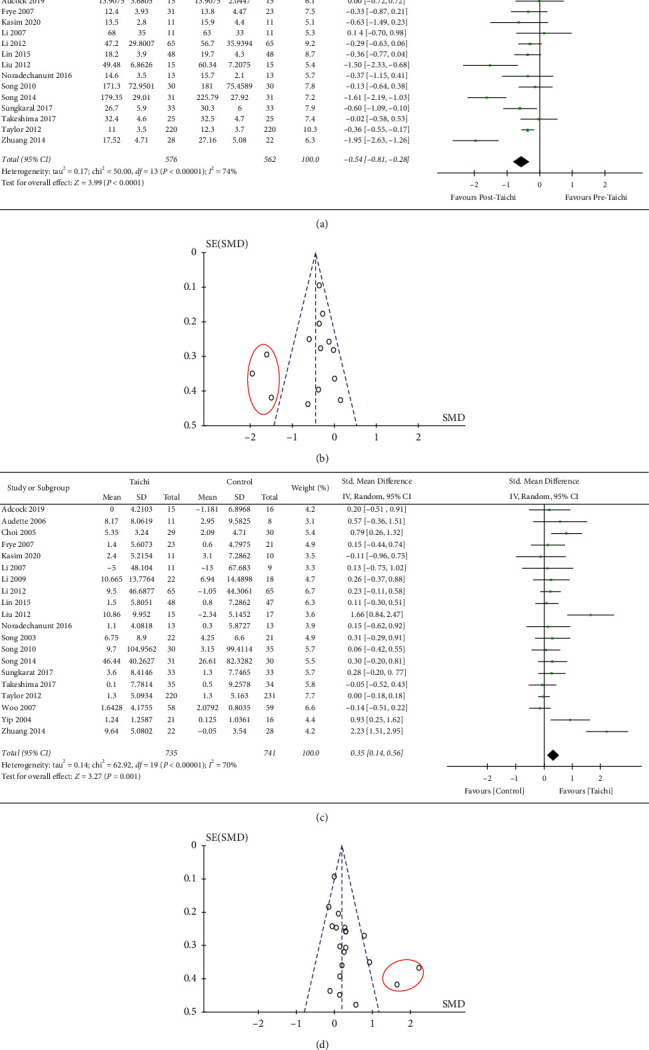
Forest plot of total data comparison. (a) Outcome of lower body strength before and after Tai Chi exercise. (b) Funnel plot of lower body strength before and after Tai Chi exercise. (c) Outcome of lower body strength in Tai Chi and control groups. (d) Funnel plot of lower body strength in Tai Chi and control groups.

**Figure 4:**
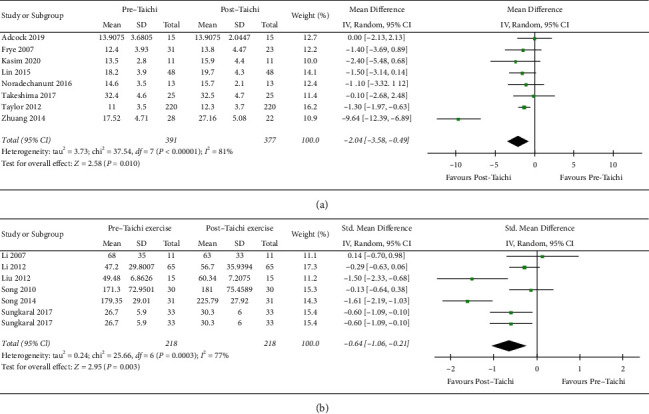
Forest plot of comparison. Subgroup outcome of forest plot in Figure 3(a). (a) 30 s chair stand test method. (b) Other test methods.

Excluding 5 trials without baseline data and 5 with incomplete data about the effect after Tai Chi exercise, we analyzed a total of 14 distinguished studies to compare the effect before and after Tai Chi exercise on lower body strength. There was statistically significant improvement in lower body strength after Tai Chi exercise with high heterogeneity by a fixed effect model. Random effect model was then adopted for analysis, and there was still highly heterogeneity in this sequence (−0.54, 95% CI [−0.81, −0.28], *p* < 0.00001, *I*^2^ = 74%) in Figure 3(a). Sensitivity analysis and subgroup analysis were then used to evaluate the following:

(1) The method of sequential exclusion from the analysis was used to evaluate whether it had an impact on the heterogeneity and outcome index. As shown in Table 2, after removing each trial in turn, the heterogeneity was still large (*p* < 0.001, *I*^2^ > 60%), which suggested that the source was not from one certain trial. (2) Three papers which may have publication bias were excluded (Liu et al. [28], Song et al. [32], Zhang et al. [19]) after being checked by funnel plot analysis as indicated in Figure 3(b). It was shown in Appendix S1 that the heterogeneity was decreased significantly (*p*=0.85, *I*^2^ = 0%). However, 2 papers (Liu et al. [28], Zhang et al. [19]) were evaluated with high quality, which might arouse doubts about the weight of publication bias. (3) As distinguished methods might contribute to the factor of great heterogeneity in Figure 3(a), subgroup analysis was carried out according to the methods of common 30 s chair stand test and other tests. After checking the 14 included trials, 8 studies were using the 30 s chair stand test method. As shown in Figures 4(a) and 4(b), based on the random effect model analysis, the trend was consistent with Figure 3(a), while it was suggested that the use of different methods (*I*^2^ = 81%, *p*=0.01; *I*^2^ = 77%, *p*=0.003) was supposed to be one of the reasons for the great heterogeneity.

To sum up, there was a significant difference in lower body strength before and after Tai Chi exercise, especially in the research of Liu et al. [28], Song et al. [32], and Zhang et al. [19]. Even though there was great heterogeneity among these 14 trials, mainly caused by publication bias analysis and subgroup analysis, the same results were obtained. Namely, Tai Chi exercise clearly increases the lower body strength of the elderly.

#### 3.4.2. Analysis of the Comparison of Tai Chi Exercise Group and Control Group in Enhancing Lower Body Strength

**Figure 5:**
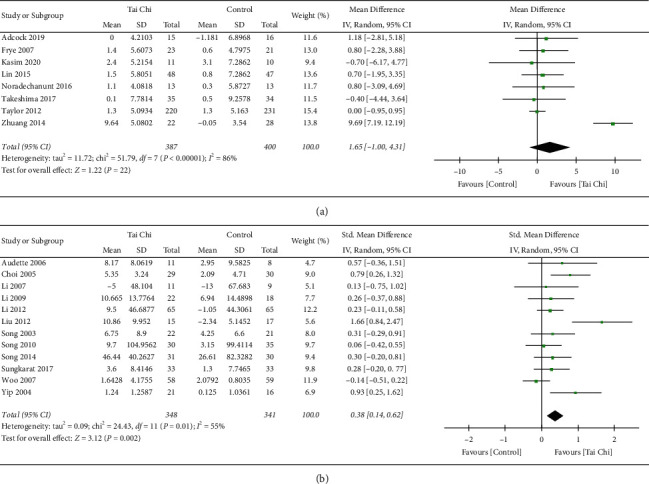
Forest plot of comparison. Subgroup outcome of forest plot in Figure 3(c). (a) 30 s chair stand test method. (b) Other test methods.

Excluding 4 studies with incomplete data, we included a total of 20 articles in the meta-analysis. As shown in Figure 3(c), there was a statistically significant difference in lower body strength between Tai Chi exercise and control group (SMD = −0.35, 95% CI [0.14, 0.56], *p*=0.001). Random effect model was adopted for analysis, and there was great heterogeneity in this sequence (*p* < 0.00001, *I*^2^ = 70%). Sensitivity analysis and subgroup analysis were also used to evaluate the potential sources of heterogeneity and the stability of the results: (1) The method of sequential exclusion from the analysis was used to evaluate whether it had an impact on the heterogeneity and outcome index. As shown in Table 3, after removing each trial, the heterogeneity was decreased (*p*=0.02, *I*^2^ = 44%), which suggested that the trial of Zhuang et al. [31] was probably one factor of high heterogeneity from the analysis. (2) As is seen in Figure 3(d), besides the trial of Zhuang et al. [31], we excluded the other trial that might also have publication bias (Liu et al. [28]). Heterogeneity decreased significantly (*p*=0.34, *I*^2^ = 10%) in Appendix S2, which suggested that the source of the greater heterogeneity in Figure 3(c) might be related to the publication bias. However, these 2 trials were evaluated with high quality, which revealed that Tai Chi exercise, compared to sedentary or low intensity exercise, indeed increased the lower body strength of the elderly. Therefore, the results of this study are relatively stable. (3) Subgroup analysis was carried out according to the methods of common 30 s chair stand test and other tests. A total of 8 articles with the 30 s chair stand test method and 12 articles with other test methods are included in Figures 5(a) and 5(b). The trend of the first subgroup (*p*=0.22) was inconsistent with the trend in Figure 3(c). It was suggested that 30 s chair stand test method (*p* < 0.00001, *I*^2^ = 86%) was more likely related to the great heterogeneity in this analysis.

To sum up, there was a significant difference in lower body strength with Tai Chi exercise, especially in the research of Zhuang et al. [31] and Liu et al. [28] which contributed vital weight but high heterogeneity to the conclusion. It revealed that the significant effects of Tai Chi exercise compared with control group should be reconsidered with high heterogeneity by containing measurements such as 30 s chair stand test.

### 3.5. Tai Chi Exercise and Knee Muscle Strength Risk

#### 3.5.1. Analysis of Tai Chi Exercise/Control Group in Knee Muscle Strength

**Figure 6:**
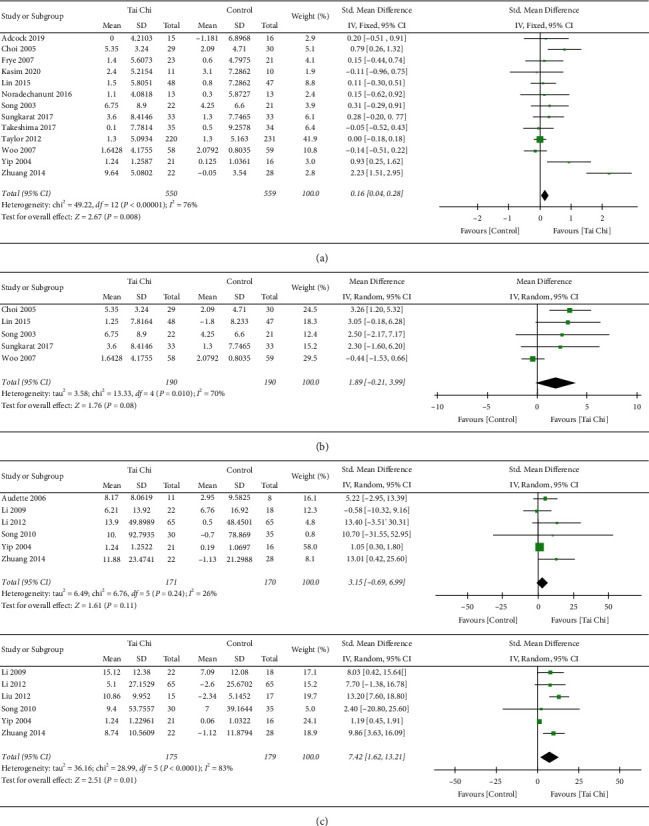
Forest plot of comparison. Outcome of Tai Chi and control knee strength. (a) Outcome of Tai Chi and control knee strength. (b) Knee strength with spring gauge test. (c) Knee extension/flexion strength with isokinetic dynamometer test.

As the effect of Tai Chi is different on different joint muscles, the results of high heterogeneity should be discussed in terms of subgroup analysis of mainly functioning muscle groups. A total of 13 studies were included in the subgroup analysis between Tai Chi exercise and control group in knee muscle strength in Figure 6(a). It was suggested that the knee muscle strength of elderly individuals with Tai Chi exercise is significantly higher than that of the control group (*p*=0.0008). Meanwhile, there was great heterogeneity (*p* < 0.00001, *I*^2^ = 76%) in this analysis, which was probably caused by the difference in sample size, measurement, and publication bias. We excluded 1 trial which had obvious publication bias (Zhuang et al. [31]) according to funnel plot analysis in Appendix S3 and showed no significant improvement to knee strength in Tai Chi exercise group compared with control group (0.10, [−0.02, 0.23], *p*=0.11). The decreased heterogeneity (*p*=0.11, *I*^2^ = 34%) indicated that the source of greater heterogeneity might be related to different outcomes of muscle groups. While Choi et al. [25], Yip et al. [27], and Zhuang et al. [31] pointed out that Tai Chi exercise evidently improved knee muscle strength compared with the control group, other included studies proved that there was no statistical difference. To sum up, Tai Chi exercise showed no significant improvement on knee muscle strength after decreasing the high heterogeneity.

#### 3.5.2. Subgroup Analysis of the Effects of Tai Chi Exercise on Lower Body Muscle Strength by Various Test Methods

*(1) Spring Gauge Test*. The spring gauge test is used to assess the maximum strength to complete resistance to evaluate the effect of exercise on overall knee muscle strength. 5 articles with this method were included in the subgroup analysis with greater heterogeneity (*p*=0.08, *I*^2^ = 70%) in Figure 6(b). This indicates that data obtained by this method may be one of the reasons for the great heterogeneity of Figure 6(a). However, after removing the data obtained by this method, the results still showed high heterogeneity (*p*=0.02, *I*^2^ = 78%), and regression analysis was needed. It also revealed that the main reason for the large heterogeneity in Figure 6(a) is the publication bias mentioned rather than different detection methods. To sum up, measuring knee strength by spring gauge test may generate heterogeneity in outcomes, which indicates that Tai Chi has no significant improvement on knee joint muscle strength.

*(2) The Isokinetic Dynamometer*. The isokinetic dynamometer test is mainly used to measure the effects of exercise on knee flexion and extension strength. The analysis of knee extension strength and knee flexion strength in Figure 6(c) is shown as follows: according to the subgroup analysis of knee muscle strength, it was found specifically that Tai Chi had no obvious effect on knee extension strength (3.15, [−0.69, 6.99], *p*=0.24, *I*^2^ = 26%) but had a significant effect on knee flexion strength (*p*=0.01, *I*^2^ = 83%). Further sensitivity analysis and regression analysis were conducted. After excluding the low quality studies, we found that there was no statistical difference in knee extension strength between the Tai Chi and control groups, and the heterogeneity (*p*=0.08, *I*^2^ = 19%) was reduced more than before. However, there is a significant difference in knee flexion strength between the Tai Chi and control groups, and the heterogeneity (10.25, [6.90, 13.61], *p* < 0.00001, *I*^2^ = 0%) was dramatically reduced. It is indicated that the reasons for heterogeneity in Figure 6(a) may be related to RB.

To sum up, different measurements may be one of the reasons for the great heterogeneity in the analysis of difference knee muscle strength between Tai Chi exercise and control group. Compared with knee extension strength, knee flexion strength by isokinetic dynamometer may generate heterogeneity in outcomes and proved that there exists statistical difference between Tai Chi and control group. Namely, while there is no significant improvement on knee extension strength, Tai Chi has a significant improvement on knee flexion strength.

## 4. Discussion

The decrease of muscle fitness, especially lower body strength, is the major inducement to affect the daily life of the elderly. We have also reached the conclusion that there is a significant improvement on the lower body strength of the elderly after Tai Chi exercise. However, the results of this meta-analysis showed that Tai Chi exercise has no significant improvement on overall knee muscle strength. The high heterogeneity in our analysis may originate from the publication bias and diversity of detective methods. Further subgroup analysis showed that although there was no significant improvement in knee extension strength, knee flexion strength was indeed improved. Sensitivity analysis showed that subgroup analysis had high homogeneity after excluding low quality literature data. Therefore, the effect of Tai Chi on lower body muscle strength of the elderly should be positive. Although Tai Chi has no obvious effect on the knee joint, it is helpful to improve the muscle fitness of knee flexion. Combined with the characteristics of Tai Chi exercise, the reason why it works might be the practice of knee/hip bending and single-leg standing which effectively increase lower body strength and optimize the normal biomechanical mechanism around knee joints [1, 33]. Therefore, Tai Chi exercise can be used as a rehabilitation treatment for training the stable deep muscle group, which effectively stimulates the deep muscle group of lower body, improves the muscle strength of knee joint, enhances the flexibility of joint, and then reduces the risk of related diseases caused by insufficient lower body strength of elderly.

Previous studies of Tai Chi exercise and muscle fitness are mainly focused on the elderly, including healthy individuals as well as chronic patients. Results of this meta-analysis showed that there are 31 studies on Tai Chi and muscle strength, mainly involving fibromyalgia, disability, KOA, Parkinson, menopause, cancer, arthritis, osteoporosis, and COPD. However, few researchers studied the overall regulation of Tai Chi on muscle strength, muscle endurance, flexibility, and other muscle fitness. Various nonrandomized controlled trials also emphasize muscle fitness and muscle strength and even deeply search for the distinguished effects of lower body muscle strength. Zhou et al. [4] found in a cross-sectional study that the muscle strength improvement degree is different in iliopsoas, quadriceps femoris, hamstring muscle, and tibial anterior muscle for elderly with 3 to 30 years of Tai Chi experience. Another evaluated method-surface electromyography of the anterior tibialis and lateral gastrocnemius muscles was recorded and showed that lower extremity muscle cocontraction plays a role in the observed benefit of longer-term Tai Chi training on gait and postural control [34]. Lu et al. [35] pointed out that at least three years of Tai Chi exercise caused different changes in knee joint strength for the elderly, particularly in extension and flexion. Tsang and Hui-Chan [36] found that the strength of knee extension (*p*=0.004), knee flexion (*p*=0.021), eccentric knee extension (*p*=0.049), and knee flexion (*p*=0.007) was significantly improved in the elderly with 3-year Tai Chi exercise. Wu et al. [37] found that only the knee extension strength of the elderly who had been practicing in Tai Chi for 3 years was significantly improved (*p* < 0.013). The result showed that the strength of quadriceps femoris in Tai Chi was significantly increased after 6 months of Tai Chi exercise, with lower body muscle strength being mainly reflected in the hip, knee, and ankle joint activities related muscle strength. Generally, muscle strength is supposed to be evaluated by isokinetic or isometric movement of related muscle groups [38]. Due to lack of relevant literature, the muscle fitness of knee joint extension and flexion is mainly selected in this study, which needs to be discussed for further research.

This study has several limitations as follows: (1) The control group does not include other types of exercise and different intensities (e.g., resistance exercise, water sports) to comprehensively evaluate the advantages and disadvantages of Tai Chi in improving lower body muscle fitness. (2) Lower quality papers should be excluded when heterogeneity occurs in the risk assessment. Even though we did not analyze all outcomes in the analysis process, relevant characteristics of these original literature were still shown in the literature feature, Table 3) We have not consider different physiology characteristics in different genders; exercise load and Tai Chi style may influence the effects on muscle fitness. In addition, the test of lower body muscle strength also tends to be replaced by other detection methods such as 3/5 chair combat strength test and heel rise test which are not included in the study. The above factors may have publication bias in the analysis of the results.

## 5. Conclusions

As a complementary and alternative rehabilitation method, Tai Chi exercise has been widely accepted by clinical researchers as well as elderly people with chronic diseases. Our study reveals that Tai Chi can improve lower body strength but has no significant effect on the knee joint-related muscle groups. Furthermore, the lower body muscle strength of the elderly decreased with age, and Tai Chi did not aggravate the declining trend. Therefore, Tai Chi exercise is likely to be used as a rehabilitation treatment for the training of stable deep muscle group, which effectively stimulates the deep muscle group of lower body, thus indirectly enhancing joint activity, improving muscle fitness, and improving functional activities of elderly individuals.

## Figures and Tables

**Table 1:** Study characteristics of the 25 included RCTs.

Study	Objects, y	Num. (pre/post)		Training program		Control	Assessment methods	Outcomes
		Tai Chi	Control	Intervention	Frequency			
Zhang et al. [19]	Elderly, ≥60	18	18	Tai Chi	16 w	—	—	Knee flexion/extension
Kasim et al. [23]	Elderly, 65∼75	11	10	Tai Chi	60 min/time, 3 times/w, 12 w	Zumba Gold	30 s chair stand test	Lower body strength
Adcock et al. [39]	Elderly, ≥65	18/15	19/16	Home training including Tai Chi-inspired exercises	30–40 min/time, 3 times/w, 16 w	—	30 s chair stand test	Lower body strength
Sungkarat et al. [29]	Amnestic mild cognitive impairment, ≥60	33	33	Tai Chi	50 min/time, 3 times/w, 15 w	—	Spring gauge test	Knee extension strength
Takeshima et al. [40]	Elderly, 67–79	35	34	Tai Chi	60 min/day, 2 days/week, 12 w	—	30 s chair stand test	Lower body strength
Noradechanunt et al. [41]	Elderly, ≥60	13	13	Tai Chi	90 min/time, 2 times/w, 12 w	—	30 s chair stand test	Lower body strength
Huang and Lin [24]	Elderly	48	47	Tai Chi	—	—	30 s chair stand test/manual muscle tester	Lower body strength/keen extension strength
Xu et al. [20]	Obese elderly women, ≥60	29	9	Tai Chi plus a behavioral weight loss program	45 min/day, 2 days/week, 16 w	—	Manual muscle dynamometer	Knee extensor torque
Guo et al. [17]	Elderly	16	9	Tai Chi	—	—	Isokinetic dynamometer test	Knee flexion/extension
Song et al. [32]	Elderly women	31	30	Tai Chi	40 min/day, 6 days/week, 12 m	Walking	Isokinetic dynamometer test	Knee extension strength
Zhuang et al. [31]	Elderly, 60–80	22	28	Combined exercise including 8-form Yang style Tai Chi	60 min/day, 3 days/week, 16 w	—	30 s chair stand test/isokinetic dynamometer test	Knee flexor extensors
Day et al. [16]	Preclinically disabled elderly, ≥70	171	190	Modified Sun style Tai Chi	60 min/day, 2 days/week, 24 w	—	30 s chair stand test/spring gauge test	Lower body strength/quad strength
Day et al. [16]	Parkinson elderly	65	65	Tai Chi	60 min/day, 2 days/week, 24 w	Stretching	Isokinetic dynamometer test	Knee extensors and flexors
Liu et al. [28]	Elderly, 60–85	15	17	Tai Chi	45 min/day, 2 days/week, 16 w	—	Biodex System 3 dynamometer	Plantar flexion and dorsiflexion
Taylor et al. [30]	Community residing elderly	220	231	Modified 10-form Sun style Tai Chi	60 min/day, 2 days/week, 20 w	Low-level exercise	30 s chair stand test	Lower limb strength
Song et al. [21]	OA elderly women	30	35	Tai Chi	55–65 min/w with instructors, 20 min/day/w by self, 6 m	—	Isokinetic dynamometer test	Knee flexor/extensor
Li et al. [5]	Community-based elderly, ≥60	22	18	24-form Tai Chi	60 min/time, 4 times/w, 6 w; 60 min/day/w, 10 w	—	Isokinetic dynamometer test	Knee flexion/extension
Frye et al. [42]	Elderly	31/23	23/21	Tai Chi	60 min/time, 3 times/w, 12 w	—	30 s chair stand test	Lower body strength
Buto et al. [3]	Community-dwelling seniors	11	9	24-form Yang style Tai Chi	60 min/time, 1 time/w, 12 m	—	Heel rise test	Lower limbs strength
Woo et al. [26]	Community-based Elderly, 65–74	30	30/29	Yang style with 24 forms of Tai Chi (TC)	3 times/w, 12 m		A quadriceps device	Strength of quadriceps
Audette et al. [22]	Elderly women, ≥65	11	8	Tai Chi	60 min/time, 3 times/w, 12 w	Brisk walking	A BTE work simulator	Knee extensor strength
Choi et al. [25]	Fall-prone elderly	29	30	12 forms of Sun style Tai Chi	35 min/time, 3 times/w, 12 w	—	Manual muscle tester	Knees extension and flexion
Yip et al. [27]	Osteoarthritis elderly	21	16	Arthritis self-management program including Tai Chi	120 min/time, 6 times/w, 16 w	—	A score of “5” assessment	Hamstring strength/quadriceps strength
Song et al. [14]	OA elderly women	22	21	12 forms of Sun style Tai Chi	20 min/time, ≥3 times/w, 12 w	—	Isokinetic dynamometer teat	Knee muscle strength
Wolf et al. [18]	Community-based elderly	72	64	Tai Chi and balance training	15 w	—	Nicholas MMT 0116 muscle tester	Hip, knee, or ankle strength

**Table 2:** Sensitivity analysis of lower body strength before and after Tai Chi exercise.

Study excluded	SMD	95% CI	*I* ^2^ (%)	*p* (heterogeneity)
Adcock et al., 2019	−0.58	−0.86, −0.30	75	<0.00001
Frye et al., 2007	−0.56	−0.85, −0.28	76	<0.00001
Kasim et al., 2020	−0.54	−0.82, −0.26	76	<0.00001
Buto et al., 2007	−0.58	−0.86, −0.31	75	<0.00001
Li et al., 2012	−0.57	−0.87, −0.28	76	<0.00001
Huang and Lin, 2015	−0.56	−0.86, −0.27	76	<0.00001
Liu et al., 2012	−0.49	−0.75, −0.23	72	<0.0001
Noradechanunt et al., 2016	−0.56	−0.84, −0.28	76	<0.00001
Song et al., 2010	−0.58	−0.87, −0.30	75	<0.00001
Song et al., 2014	−0.45	−0.69, −0.21	64	0.0008
Sungkarat et al., 2017	−0.54	−0.83, −0.25	76	<0.00001
Takeshima et al., 2017	−0.59	−0.87, −0.31	75	<0.00001
Taylor et al., 2012	−0.57	−0.90, −0.25	75	<0.00001
Zhuang et al., 2014	−0.67, −0.22	−0.67, −0.22	61	0.002

**Table 3:** Sensitivity analysis of lower body strength between Tai Chi exercise and control group.

Study excluded	SMD	95% CI	*I* ^2^ (%)	*p* (heterogeneity)
Adcock et al., 2019	0.36	0.14, 0.58	71	<0.00001
Audette et al., 2006	0.34	0.13, 0.56	71	<0.00001
Choi et al., 2005	0.32	0.11, 0.54	69	<0.00001
Frye et al., 2007	0.36	0.14, 0.58	71	<0.00001
Kasim et al., 2020	0.37	0.15, 0.58	71	<0.00001
Buto et al., 2007	0.36	0.14, 0.58	71	<0.00001
Li et al., 2009	0.36	0.14, 0.58	71	<0.00001
Li et al., 2012	0.36	0.14, 0.59	71	<0.00001
Huang and Lin, 2015	0.37	0.15, 0.60	71	<0.00001
Liu et al., 2012	0.29	0.10, 0.49	64	<0.0001
Noradechanunt et al., 2016	0.36	0.14, 0.58	71	<0.00001
Song et al., 2003	0.35	0.13, 0.57	71	<0.00001
Song et al., 2010	0.37	0.15, 0.59	71	<0.00001
Song et al., 2014	0.36	0.13, 0.58	71	<0.00001
Sungkarat et al., 2017	0.36	0.14, 0.58	71	<0.00001
Takeshima et al., 2017	0.38	0.16, 0.60	71	<0.00001
Taylor et al., 2012	0.38	0.15, 0.62	68	<0.00001
Woo et al., 2007	0.39	0.17, 0.61	70	<0.00001
Yip et al., 2004	0.32	0.11, 0.53	69	<0.00001
Zhuang et al., 2014	0.24	0.08, 0.39	44	0.02

## Data Availability

The data we used to support the findings of this study can be accessed from the paper by searching the database according to our protocol which is elaborated clearly in the manuscript.

## Supplementary Materials

Supplementary Materials The fixed forest plots of lower body strength before and after Tai Chi exercise, lower body strength in Tai Chi and control groups, and knee strength in Tai Chi and control groups are shown, respectively, in Appendices S1–3. The heterogeneity in Appendix S1 was decreased significantly (*I*^2^ = 0%, *p*=0.85). However, 2 trials (Liu et al. [28], Zhang et al. [19]) were evaluated with high quality, which aroused doubts about the weight of publication bias. In Appendix S2, the heterogeneity decreased significantly (*p*=0.34, *I*^2^ = 10%), which suggested that the source of greater heterogeneity in Figure 3(c) might be related to the publication bias. One trial with obvious publication bias (Zhuang et al. [31]) was excluded in Appendix S3; it showed no significant improvement to knee strength in Tai Chi exercise group compared with control group (*p*=0.09).

## References

[1] Zeng L., Yang W., Guo D. (2018). Systematic evaluation of the effect of traditional exercise therapy on pain improvement and joint function in knee osteoarthritis patients. *Chinese Journal of Chinese Medicine*.

[2] Zou L., Xiao T., Cao C. (2021). Tai chi for chronic illness management: synthesizing current evidence from meta-analyses of randomized controlled trials. *The American Journal of Medicine*.

[3] Buto M. S. D. S., de Oliveira M. P. B., Carvalho C., Vassimon-Barroso V., Takahashi A. C. d. M. (2020). Effect of complementary therapies on functional capacity and quality of life among prefrail and frail older adults: a systematic review of randomized controlled trials. *Archives of Gerontology and Geriatrics*.

[4] Zhou M., Peng N., Dai Q., Li H.-W., Shi R.-G., Huang W. (2016). Effect of tai chi on muscle strength of the lower extremities in the elderly. *Chinese Journal of Integrative Medicine*.

[5] Li J. X., Xu D. Q., Hong Y. (2009). Changes in muscle strength, endurance, and reaction of the lower extremities with tai chi intervention. *Journal of Biomechanics*.

[6] Yang F., Liu W. (2020). Biomechanical mechanism of tai-chi gait for preventing falls: a pilot study. *Journal of Biomechanics*.

[7] Charron S., McKay K. A., Tremlett H. (2018). Physical activity and disability outcomes in multiple sclerosis: a systematic review (2011–2016). *Multiple Sclerosis and Related Disorders*.

[8] Zou L., Wang H., Xiao Z. (2017). Tai chi for health benefits in patients with multiple sclerosis: a systematic review. *PLoS One*.

[9] Li J. X., Hong Y., Chan K. M. (2001). Tai chi: physiological characteristics and beneficial effects on health. *British Journal of Sports Medicine*.

[10] Lee A. C., Harvey W. F., Han X. (2018). Pain and functional trajectories in symptomatic knee osteoarthritis over up to 12 weeks of exercise exposure. *Osteoarthritis and Cartilage*.

[11] Solianik R., Mickevičienė D., Žlibinaitė L., Čekanauskaitė A. (2021). Tai chi improves psychoemotional state, cognition, and motor learning in older adults during the COVID-19 pandemic. *Experimental Gerontology*.

[12] Pan X., Huang L., Lv J., Wu X., Niu W., Liu Y. (2017). Effect of new type taijiquan on lower limb muscle strength and dynamic balance in elderly women with knee osteoarthritis. *Sports Science and Technology*.

[13] Huang L. (2015). *Effect of Taijiquan Intervention on Clinical Rehabilitation and Gait Biomechanics in Elderly Patients with Knee Osteoarthritis*.

[14] Song R., Lee E. O., Lam P., Bae S. C (2003). Effects of tai chi exercise on pain, balance, muscle strength, and perceived difficulties in physical functioning in older women with osteoarthritis: a randomized clinical trial. *Journal of Rheumatology*.

[15] Chen Y.-W., Hunt M. A., Campbell K. L., Peill K., Reid W. D. (2016). The effect of tai chi on four chronic conditions-cancer, osteoarthritis, heart failure and chronic obstructive pulmonary disease: a systematic review and meta-analyses. *British Journal of Sports Medicine*.

[16] Day L., Hill K. D., Jolley D., Cicuttini F., Flicker L., Segal L. (2012). Impact of tai chi on impairment, functional limitation, and disability among preclinically disabled older people: a randomized controlled trial. *Archives of Physical Medicine and Rehabilitation*.

[17] Guo L.-Y., Yang C.-P., You Y.-L. (2014). Underlying mechanisms of tai-chi-chuan training for improving balance ability in the elders. *Chinese Journal of Integrative Medicine*.

[18] Wolf S. L., Barnhart H. X., Kutner N. G., Mcneely E., Coogler C., Xu T. (2003). Selected as the best paper in the 1990s: reducing frailty and falls in older persons: an investigation of tai chi and computerized balance training. *Journal of the American Geriatrics Society*.

[19] Zhang T., Mao M., Sun W. (2021). Effects of a 16-week tai chi intervention on cutaneous sensitivity and proprioception among older adults with and without sensory loss. *Research in Sports Medicine*.

[20] Xu F., Letendre J., Bekke J. (2015). Impact of a program of tai chi plus behaviorally based dietary weight loss on physical functioning and coronary heart disease risk factors: a community-based study in obese older women. *Journal of Nutrition in Gerontology and Geriatrics*.

[21] Song R., Roberts B. L., Lee E.-O., Lam P., Bae S.-C. (2010). A randomized study of the effects of t’ai chion muscle strength, bone mineral density, and fear of falling in women with osteoarthritis. *Journal of Alternative & Complementary Medicine*.

[22] Audette J. F., Jin Y. S., Newcomer R., Stein L., Duncan G., Frontera W. R. (2006). Tai chi versus brisk walking in elderly women. *Age and Ageing*.

[23] Kasim N. F., Veldhuijzen van Zanten J., Aldred S. (2020). Tai chi is an effective form of exercise to reduce markers of frailty in older age. *Experimental Gerontology*.

[24] Huang Y., Liu X. (2015). Improvement of balance control ability and flexibility in the elderly tai chi chuan (TCC) practitioners: a systematic review and meta-analysis. *Archives of Gerontology and Geriatrics*.

[25] Choi J. H., Moon J.-S., Song R. (2005). Effects of Sun-style tai chi exercise on physical fitness and fall prevention in fall-prone older adults. *Journal of Advanced Nursing*.

[26] Woo J., Hong A., Lau E., Lynn H. (2007). A randomised controlled trial of tai chi and resistance exercise on bone health, muscle strength and balance in community-living elderly people. *Age and Ageing*.

[27] Yip Y., Sit J., Wong D. (2004). A quasi-experimental study on improving arthritis self-management for residents of an aged people’s home in Hong Kong. *Psychology Health & Medicine*.

[28] Liu J., Wang X. Q., Zheng J. J. (2012). Effects of tai chi versus proprioception exercise program on neuromuscular function of the ankle in elderly people: a randomized controlled trial. *Evidence-Based Complementary and Alternative Medicine*.

[29] Sungkarat S., Boripuntakul S., Chattipakorn N., Watcharasaksilp K., Lord S. R. (2017). Effects of tai chi on cognition and fall risk in older adults with mild cognitive impairment: a randomized controlled trial. *Journal of the American Geriatrics Society*.

[30] Taylor D., Hale L., Schluter P. (2012). Effectiveness of tai chi as a community-based falls prevention intervention: a randomized controlled trial. *Journal of the American Geriatrics Society*.

[31] Zhuang J., Huang L., Wu Y., Zhang Y. (2014). The effectiveness of a combined exercise intervention on physical fitness factors related to falls in community-dwelling older adults. *Clinical Interventions in Aging*.

[32] Song Q. H., Zhang Q. H., Xu R. M. (2014). Effect of tai-chi exercise on lower limb muscle strength, bone mineral density and balance function of elderly women. *International Journal of Clinical and Experimental Medicine*.

[33] Roig M., O’Brien K., Kirk G. (2009). The effects of eccentric versus concentric resistance training on muscle strength and mass in healthy adults: a systematic review with meta-analysis. *British Journal of Sports Medicine*.

[34] Wayne P. M., Gow B. J., Hou F. (2021). Tai chi training’s effect on lower extremity muscle co-contraction during single- and dual-task gait: cross-sectional and randomized trial studies. *PLoS One*.

[35] Lu X., Hui-Chan C. W., Tsang W. W. (2013). Tai chi, arterial compliance, and muscle strength in older adults. *European Journal of Preventive Cardiology*.

[36] Tsang W. W. N., Hui-Chan C. W. Y. (2005). Comparison of muscle torque, balance, and confidence in older tai chi and healthy adults. *Medicine & Science in Sports & Exercise*.

[37] Wu G., Zhao F., Zhou X., Wei L. (2002). Improvement of isokinetic knee extensor strength and reduction of postural sway in the elderly from long-term tai chi exercise. *Archives of Physical Medicine and Rehabilitation*.

[38] Lan C., Lai J.-S., Chen S.-Y., Wong M.-K. (2000). Tai chi chuan to improve muscular strength and endurance in elderly individuals: a pilot study. *Archives of Physical Medicine and Rehabilitation*.

[39] Adcock M., Fankhauser M., Post J. (2019). Effects of an in-home multicomponent exergame training on physical functions, cognition, and brain volume of older adults: a randomized controlled trial. *Frontiers of Medicine*.

[40] Takeshima N., Islam M. M., Kato Y. (2017). Effects of 12 weeks of tai chi chuan training on balance and functional fitness in older Japanese adults. *Sports*.

[41] Noradechanunt C., Worsley A., Groeller H. (2016). Thai yoga improves physical function and well-being in older adults: a randomised controlled trial. *Journal of Science and Medicine in Sport*.

[42] Frye B., Scheinthal S., Kemarskaya T., Pruchno R. (2007). Tai chi and low impact exercise: effects on the physical functioning and psychological well-being of older people. *Journal of Applied Gerontology*.

